# Supplementary material for: Reduction of precious metal ions in aqueous solutions by contact-electro-catalysis
Source: Nat Commun. 2024 May 17;15:4196. doi: 10.1038/s41467-024-48407-w (PMC11101412; doi:10.1038/s41467-024-48407-w)
Supplement: Supplementary file 1 — Supplementary Information [file 41467_2024_48407_MOESM1_ESM.pdf]

Supplementary Information for

# Reduction of precious metal ions in aqueous solutions by contact-electro-catalysis

Yusen Su,[1,2,10], Andy Berbille[1,2,10], Xiao-Fen Li[3,1,10], Jinyang Zhang[1,2,10], MohammadJavad PourhosseiniAsl[1,4], Huifan Li[1,5], Zhanqi Liu[1,6], Shunning Li[7], Jianbo Liu[3], Laipan Zhu[1,2,*], Zhong Lin Wang[1,2,8,9,*]

[1] CAS Center for Excellence in Nanoscience, Beijing Institute of Nanoenergy and Nanosystems, Chinese Academy of Sciences, Beijing 101400, China

[2] School of Nanoscience and Technology, University of Chinese Academy of Sciences, Beijing 100049, China

[3] China Key Laboratory of Advanced Materials (MOE), School of Materials Science and Engineering, Tsinghua University, Beijing, 100084, China

[4]Department of Materials Science and Engineering, College of Engineering, Peking University, 100871, Beijing, China

[5] Center on Nanoenergy Research, School of Physical Science and Technology, Guangxi University, Nanning 530004, China

[6] School of Physical Science and Technology, Lanzhou University, Lanzhou 730000, China

[7] School of Advanced Materials, Shenzhen Graduate School, Peking University, Shenzhen, 518055, China

[8] Yonsei Frontier Lab, Yonsei University, Seoul 03722, Republic of Korea

[9]School of Materials Science and Engineering, Georgia Institute of Technology, Atlanta, GA 30332-0245, USA

[10] These authors contributed equally.

**Corresponding authors. Email: zhong.wang@mse.gatech.edu (Z.L.W.); zhulaipan@binn.cas.cn (L.Z.)

**The PDF file includes:**

Supplementary Note 1. Details of Contact-electro-catalysis.

Supplementary Fig. 1 | Evolution of Au concentration in conditions of various radical scavengers. Ter-butanol, p-benzoquinone, DMSO, and EDTA-2Na, are regarded as superoxide radical, hydroxide radical, electron, and proton scavengers, respectively.

Supplementary Fig. 2 | Intermittent and continuous reaction in 120 W ultrasonication.

Supplementary Fig. 3 | BET isotherm plots for nitrogen adsorption capacity of 2 μm FEP. The insert is pore distribution.

Supplementary Fig. 4 | Optimization of the reaction conditions. The influence of FEP mass (a), temperature (b), NaCl concentration (c), and pH (d) on the reduction of the $AuCl_4^-$.

Supplementary Fig. 5 | Experiments with FEP powder of varying diameter. The size distribution (a) and gold extraction amount (b) of 0.2, 2, 6.5, 15, and 30 μm FEP powder.

Supplementary Fig. 6 | AuCl4- reduction by CEC in DMSO, acetonitrile, ethanol, and water.

Supplementary Fig. 7 | Reduction of 0.1 mM $AuCl_4^-$ to which 100 ppb of various additives, including SiO2, BHT (2,6-Di-tert-butyl-4-methylphenol), DIP (di(2-ethylhexyl)phthalate), in the presence of 10 mg FEP after one hour.

Supplementary Fig. 8 | Evolution of Au concentration in various concentrations including (a) 0.001 mM, (b) 0.01 mM, (c) 0.1 mM, and (d) 1 mM.

Supplementary Fig. 9 | Scanning electron microscopy of FEP before (a) and after (b) reaction.

Supplementary Fig. 10 | Energy dispersive spectrometer images (a) and spectra (b) of FEP after reaction.

Supplementary Fig. 11 | Size distribution of FEP before (a) and after (b) the reaction.

Supplementary Fig. 12 | X-ray photoelectron spectroscopy for FEP before (a) and after (b) reaction.

Supplementary Fig. 13 | Fourier Transform Infrared spectra of catalysis before and after the reaction.

Supplementary Fig. 14 | Transmission electron microscopy images for Au dispersed in water (a) and ethanol (b).

Supplementary Fig. 15 | Energy dispersive X-ray spectroscopy images of reduced Au.

Supplementary Fig. 16 | XPS Au4*f* spectra of reduced gold.

Supplementary Fig. 17 | Energy dispersive X-ray spectroscopy images of reduced Pd (a), Pt (b), Ag (c), Rh (d), and Ir (e).

Supplementary Fig. 18 | Calculated distance between O of oxygen and F, and adsorption energy for PTFE and $O_2$. Carbon: Purple, Grey: Fluoride, Red: Oxygen, Yellow: Gold.

Supplementary Fig. 19 | Simulated energy difference ΔE(HOMO(PTFE)- π(O2)) for isotactic, syndiotactic and atactic PP. Carbon: purple, Hydrogen: blue, Oxygen: red.

Supplementary Fig. 20 | Real world application for electroplating waste.

Supplementary Fig. 21 | Fe, Ni, Cu, Zn, and Au extraction amount in their anaerobic aqueous solution.

Supplementary Fig. 22 | GPC analysis of pristine PP. Mn=62672, Mw=289392, Mz=755130.

Supplementary Fig. 23 | TG-DSC analysis of pristine (a) FEP, (b) HDPE, (c) PP, and (d) PTFE.

Supplementary Fig. 24 | Fourier Transformed Infra-Red Spectroscopy (FTIR) of pristine (a) FEP, (b) HDPE, (c) PP, and (d) PTFE.

Supplementary Fig. 25 | Photograph of reactor. The seal consists of a PTFE gasket, rubber ring and silica gel grommet.

Supplementary Table 1. Intermittent and continuous result for 120 W 40 kHz and 60 W 40kHz ultrasonication.

Supplementary Table 2. Surface properties of different sizes of FEP powder.

Supplementary Table 3. Comparisons between representative metal reduction literatures and this work.

Supplementary Table 4. D-spacing and SAED analysis of reduced metals.

## Supplementary Note 1: Details of Contact-electro-catalysis

The principle of contact-electro-catalysis is based on the exchange electrons occurring when water contacts the surface of a dielectric insulator[1]. When a water molecule contacts a polymer the electron clouds of the solid and the liquid could overlap[2]. At this point, if the electron affinity of the polymer is sufficiently high, then 1 electron can be transferred from the molecule of water to the polymer chain (**Equation 1**). Thereafter the water radical cation thus formed can react with a water molecule and for a hydronium cation and a hydroxyl radical (**Equation 2**), and the polymer is now charged (symbolized by an asterisk in **Equation 1**)[3].

$$(1)\ H_2O_{(l)} + FEP_{(s)} \xrightarrow{SL-CE} H_2O^{\bullet+}_{(aq)} + FEP^*_{(s)}$$

$$(2)\ H_2O^{\bullet+}_{(aq)} + H_2O_{(l)} \rightarrow H_3O^{+}_{(aq)} + HO^{\bullet}_{(aq)}$$

Under ultrasonic conditions[4], or if the material is exposed to visible light[5], there is a probability that the electron gets excited from the surface of the charge polymer towards the bulk of water (**Equation 3**)[3]. This electron can then react with $O_2$ gas dissolved in the solution to form superoxide radicals (**Equation 4**)[3].

$$(3)\ FEP^*_{(s)} \xrightarrow[heat]{photons,\ phonons,} e^- + FEP_{(s)}$$

$$(4)\ O_{2(g)} + e^- \rightarrow O^{\bullet-}_{2(aq)}$$

Under ultrasonic conditions, the contact-separation cycle that allows for the exchange of electrons by contact-electrification between water and the dielectric insulator (catalyst) is provided by the high frequency generation of microbubbles in the vicinity of or at the surface of the catalyst[6]. When the bubble grows at/near the surface of the solid, it chasses the water while the electrons are ejected from the surface upon absorption of phonon, heat or light[3]. When the bubble collapses, not only does the water contacts the solid again, which allows for 1 more cycle of electron transfers, but it may do it with a high force or velocity owing to the formation of a high pressure microjet[3]. As long as the material and aqueous solutions are exposed to ultrasonication, the catalytic cycle continues. In this case, a high number of electrons are generated, as illustrated by the EPR data reported in **Fig. 1d** in the main text. In this case, it is possible to reduce the metals metal ions mentioned in the main text (**Equation 5**).

$$(5)\ M^{n+}_{(aq)} + ne^- \rightarrow M^0_{(s)}$$

It is worth noting other mechanisms may contribute to effects of CEC, including the contribution of the

corona of bubbles on solid surface[7], or exchange of hydroxyl functional groups between water and the surface of $SiO_2$[8].

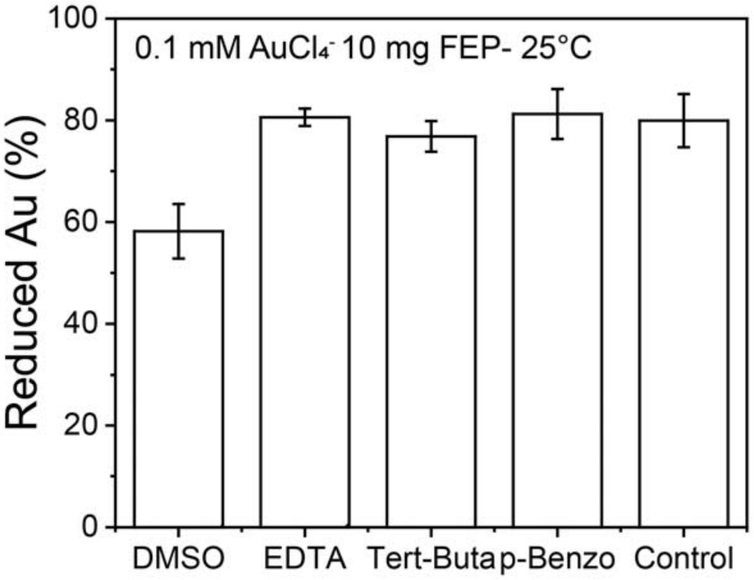


**Supplementary Fig. 1 |** Evolution of Au concentration in conditions of various radical scavengers. Ter-butanol, p-benzoquinone, DMSO, and EDTA-2Na, are regarded as superoxide radical, hydroxide radical, electron, and proton scavengers, respectively. For all figures, error bars represent standard deviations for 3 reproduced experiments.

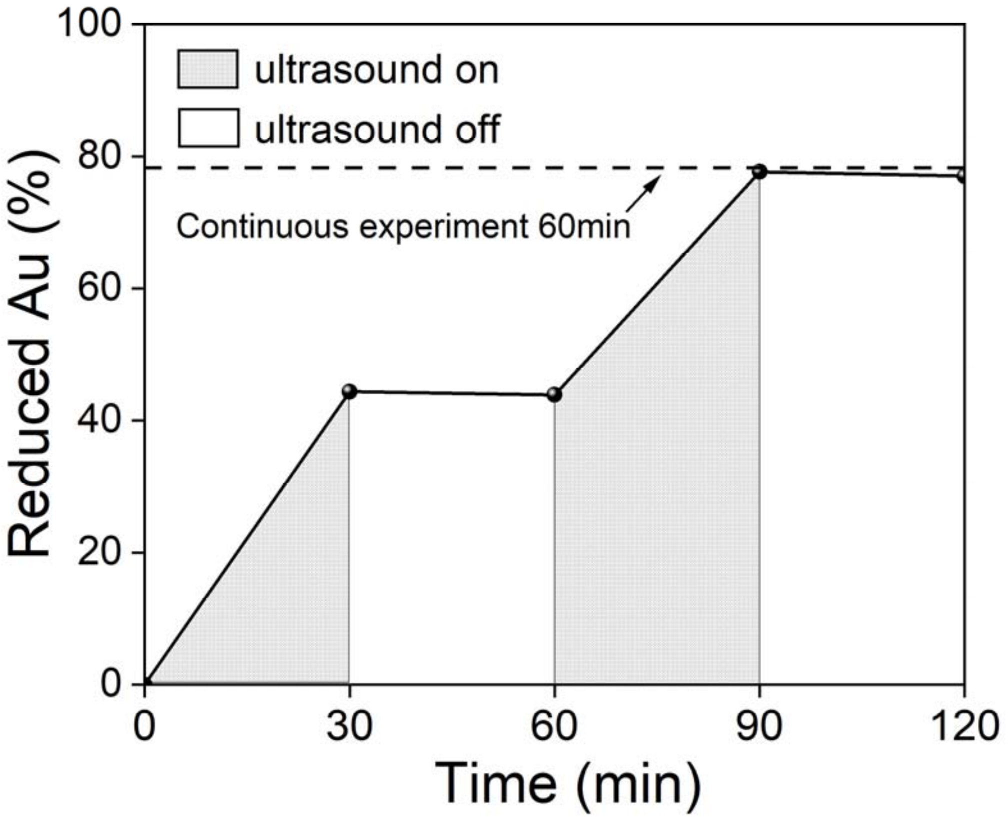


**Supplementary Fig. 2 |** Intermittent and continuous reaction in 120 W ultrasonication.

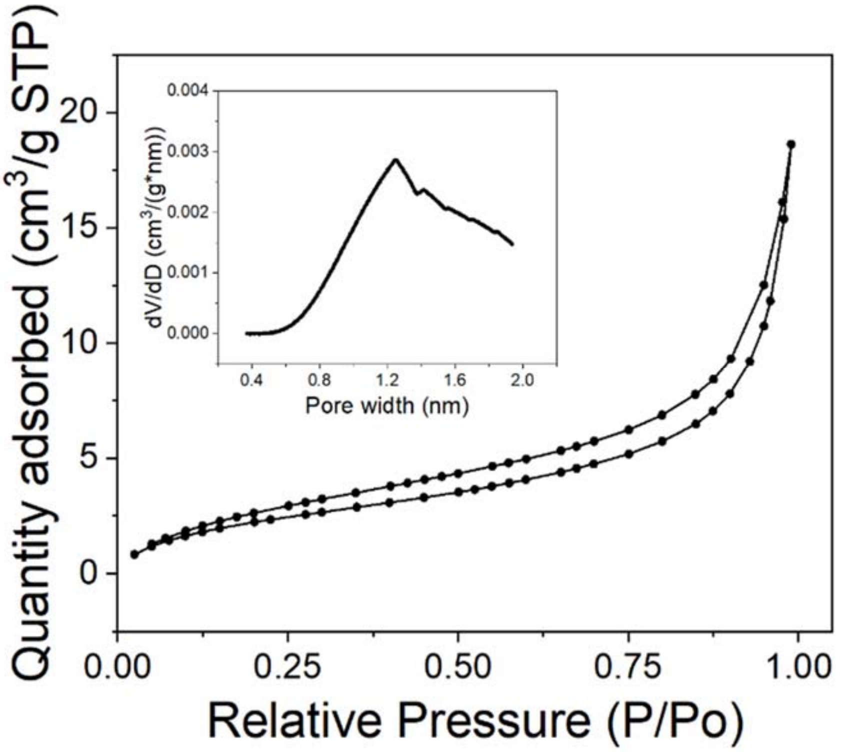


**Supplementary Fig. 3 |** BET isotherm plots for nitrogen adsorption capacity of 2 μm FEP. The insert is the pore distribution.

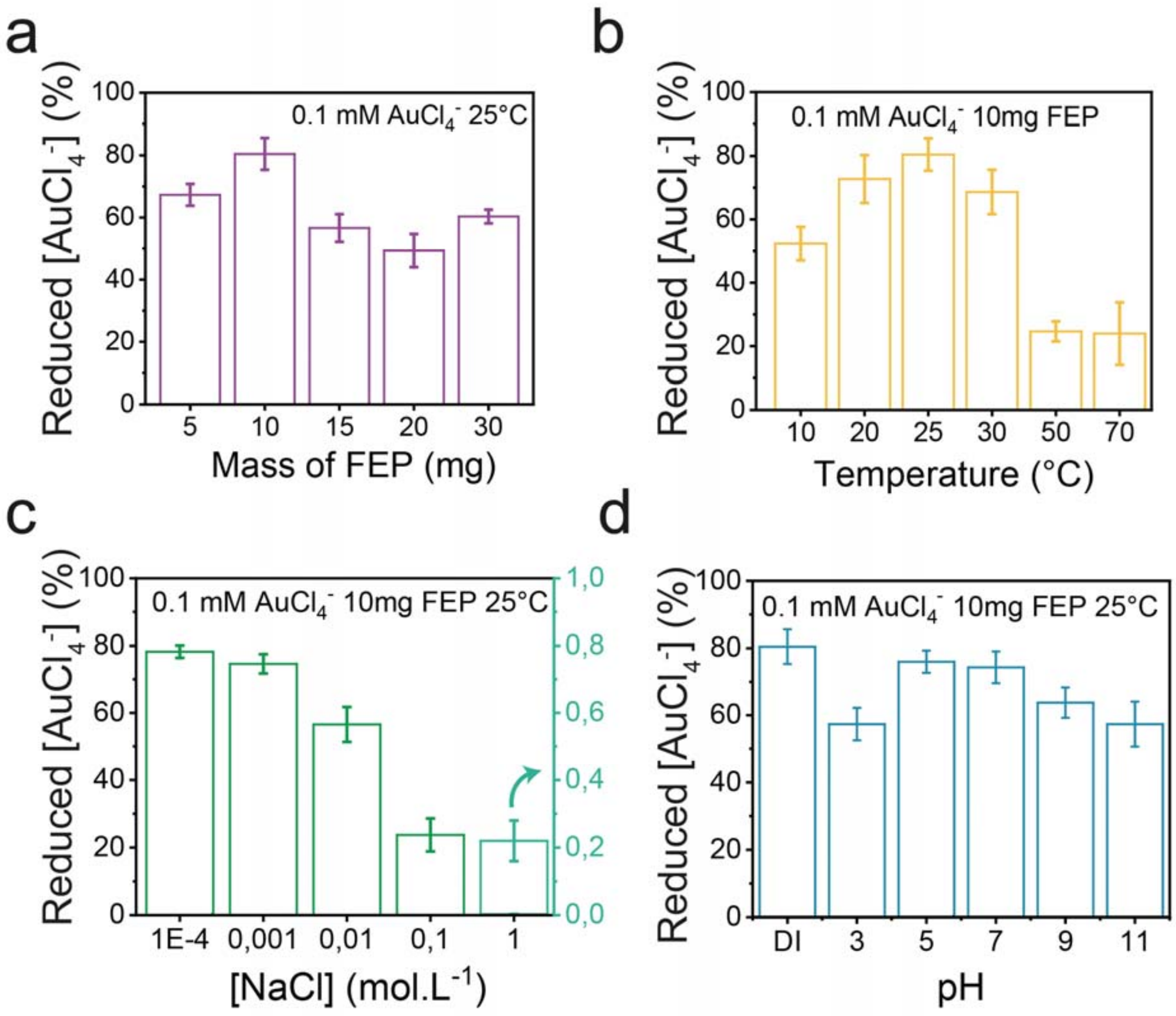


**Supplementary Fig. 4 |** Optimization of the reaction conditions. The influence of FEP (a) mass, (b) temperature, (b) NaCl concentration, and (d) pH on the reduction of the $AuCl_4^-$. For all figures, error bars represent standard deviations for 3 reproduced experiments.

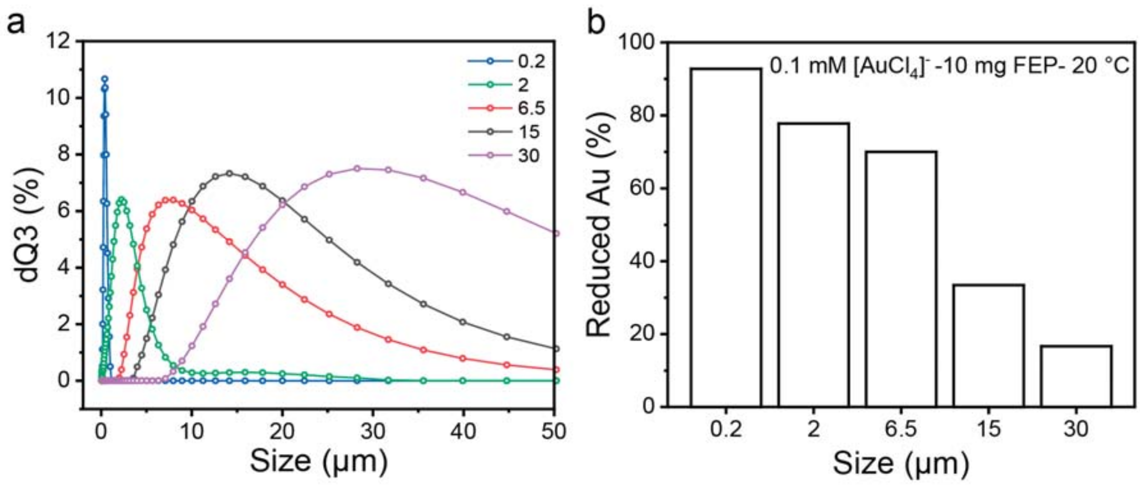


**Supplementary Fig. 5 |** Experiments with FEP powder of varying diameter. The size distribution (a) and gold extraction amount (b) of 0.2, 2, 6.5, 15, and 30 μm FEP powder.

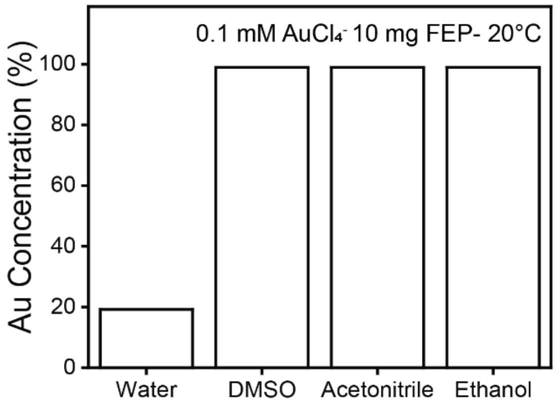


**Supplementary Fig. 6 |** $AuCl_4^-$ reduction by CEC in DMSO, acetonitrile, ethanol, and water.

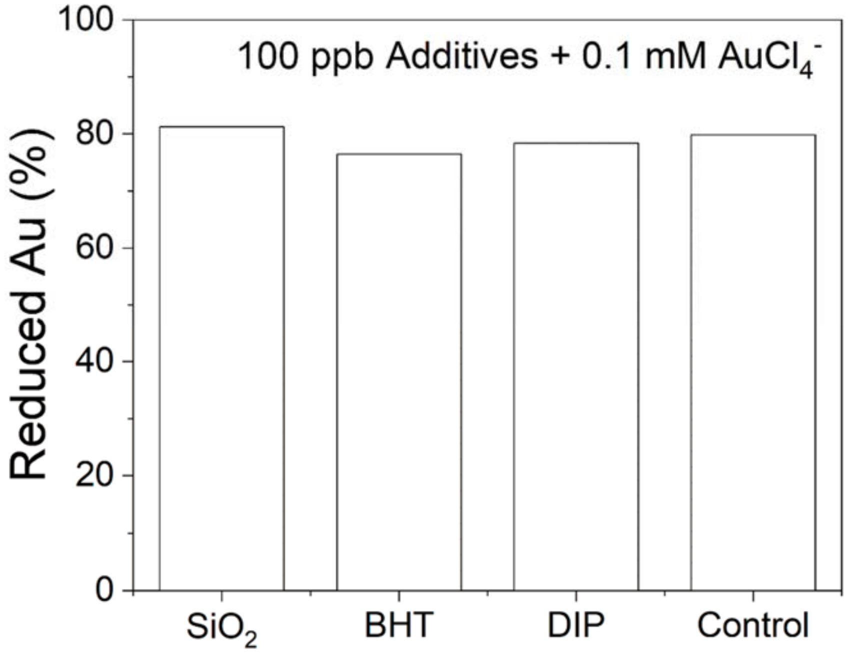


**Supplementary Fig. 7 |** Reduction of 0.1 mM $AuCl_4^-$ to which 100 ppb of various additives, including $SiO_2$, BHT (2,6-Di-tert-butyl-4-methylphenol), DIP (di(2-ethylhexyl)phthalate), in the presence of 10 mg FEP after one hour. The control is a standard experiment in absence of any additive in the solution.

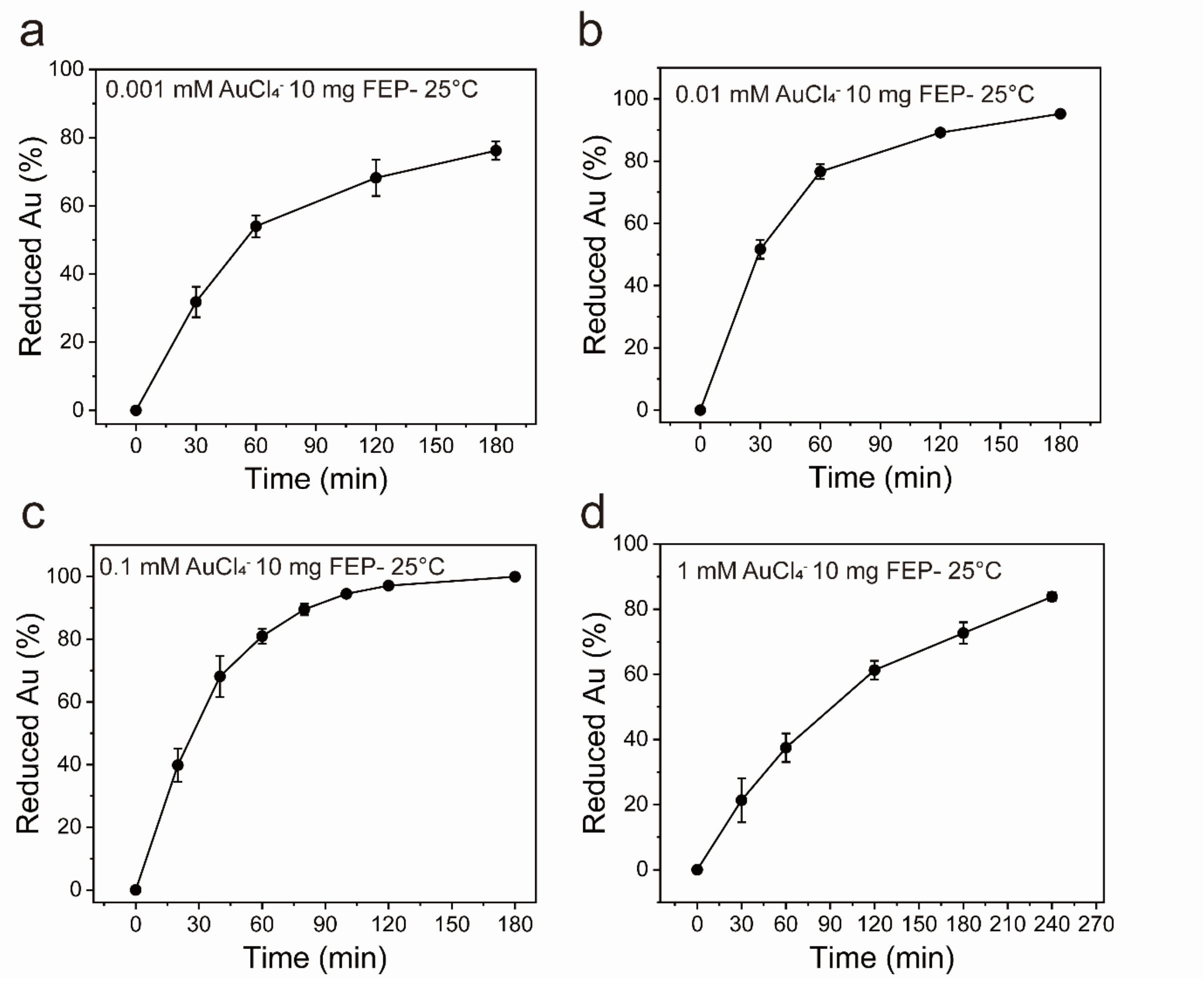


**Supplementary Fig. 8 |** Evolution of Au concentration in various concentrations including (a) 0.001 mM, (b) 0.01 mM, (c) 0.1 mM, and (d) 1 mM. Lines are guide to eyes. For all figures, error bars represent standard deviations for 3 reproduced experiments.

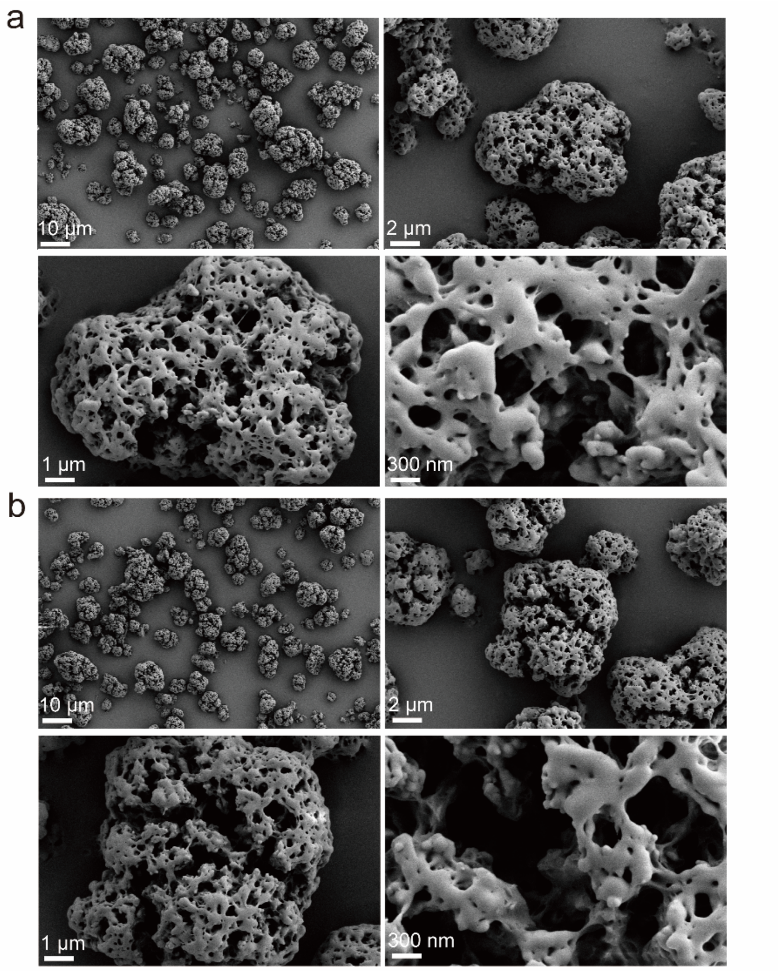


**Supplementary Fig. 9 |** Scanning electron microscopy of FEP (a) before and (b) after (b) reaction.

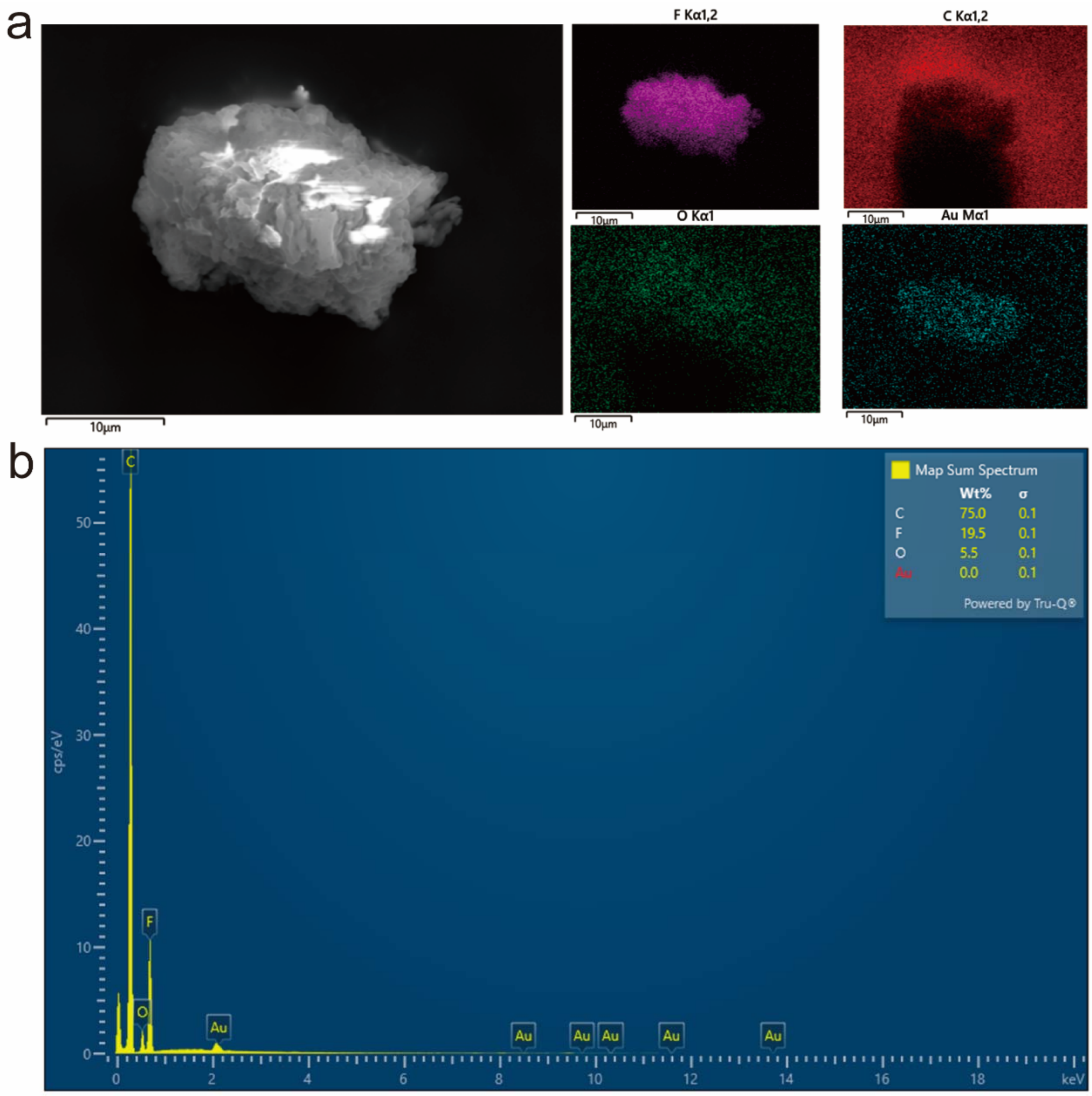


**Supplementary Fig. 10 |** (a) Energy dispersive spectrometer images and (b) spectrum of FEP after reaction.

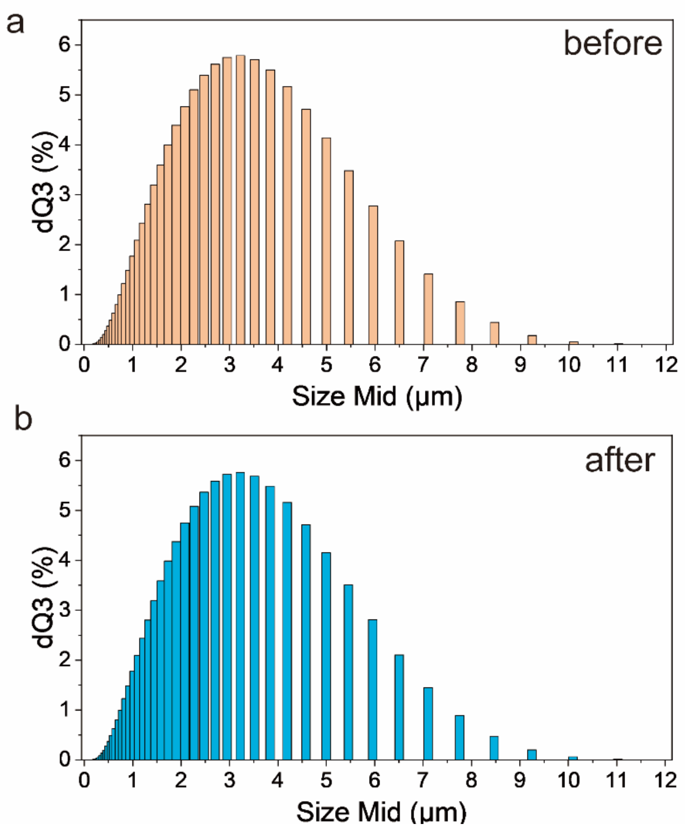


**Supplementary Fig. 11 |** Size distribution of FEP (a) before and (b) after the reaction.

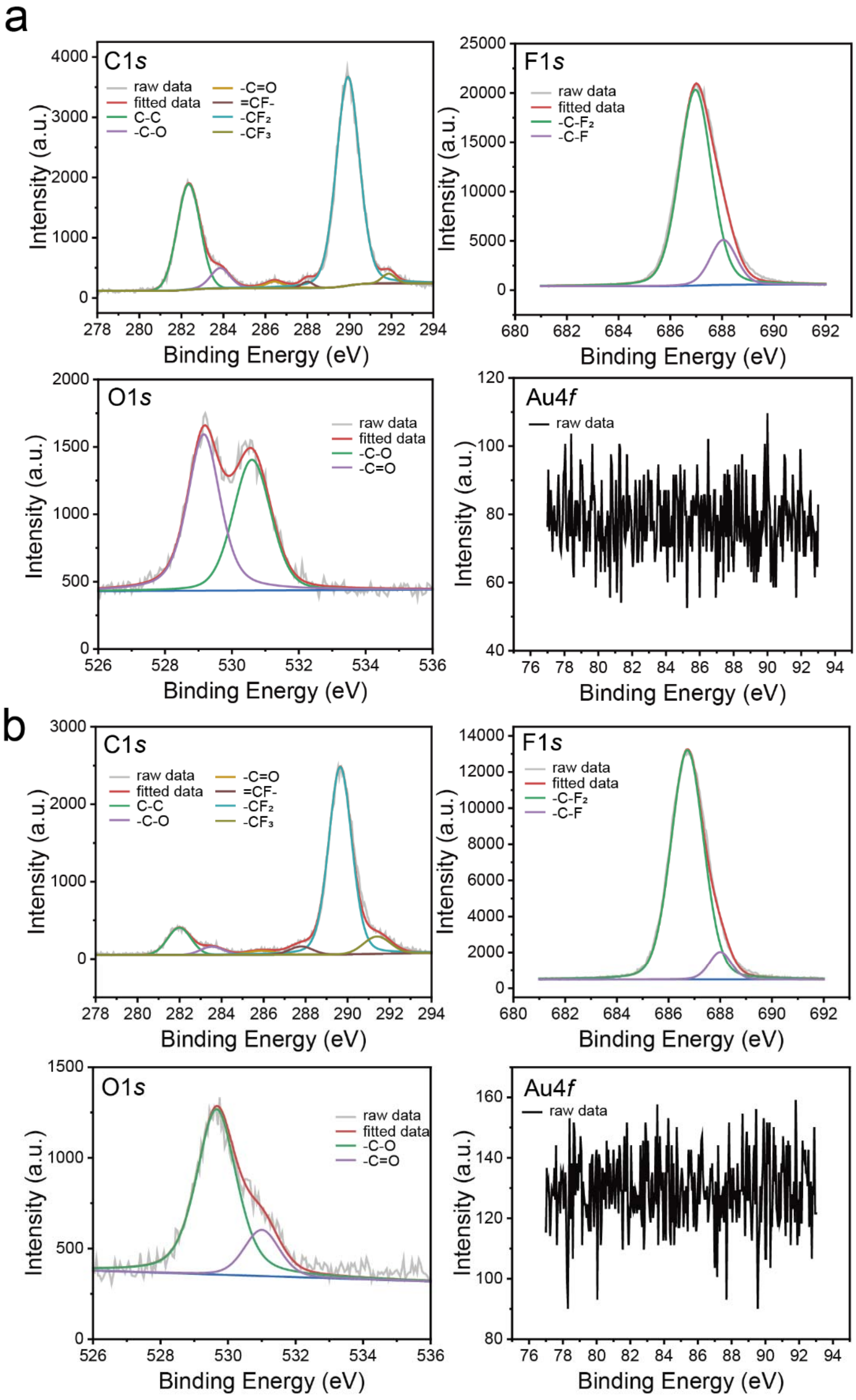


**Supplementary Fig. 12 |** X-ray photoelectron spectroscopy for FEP (a) before and (b) after reaction.

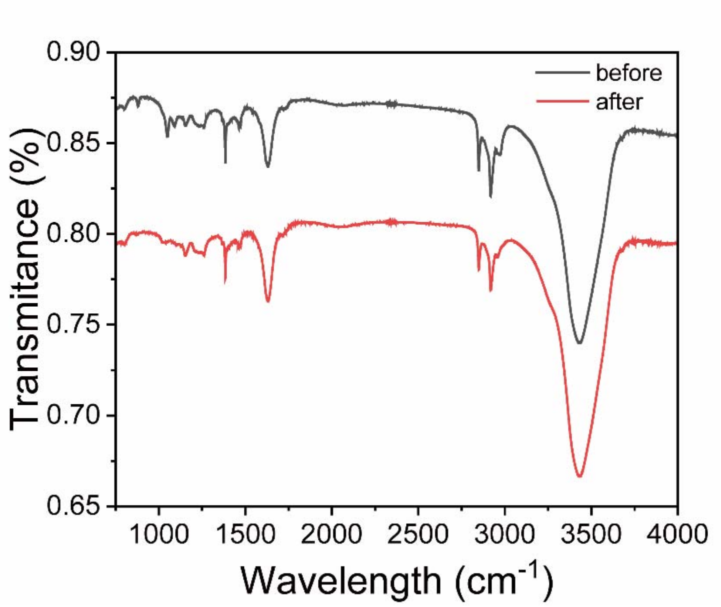


**Supplementary Fig. 13 |** Fourier Transform Infrared spectra of catalysis before and after the reaction.

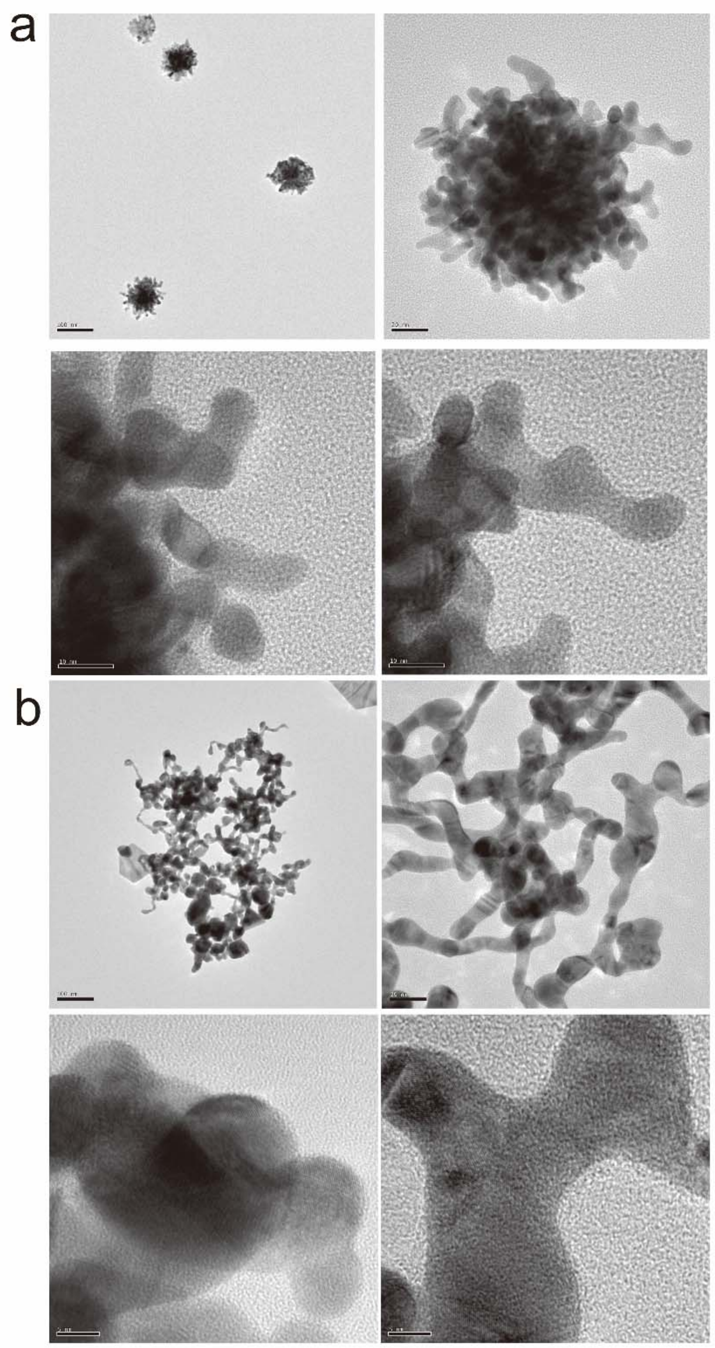


**Supplementary Fig. 14 |** Transmission electron microscopy images for Au dispersed in (a) water and (b) ethanol.

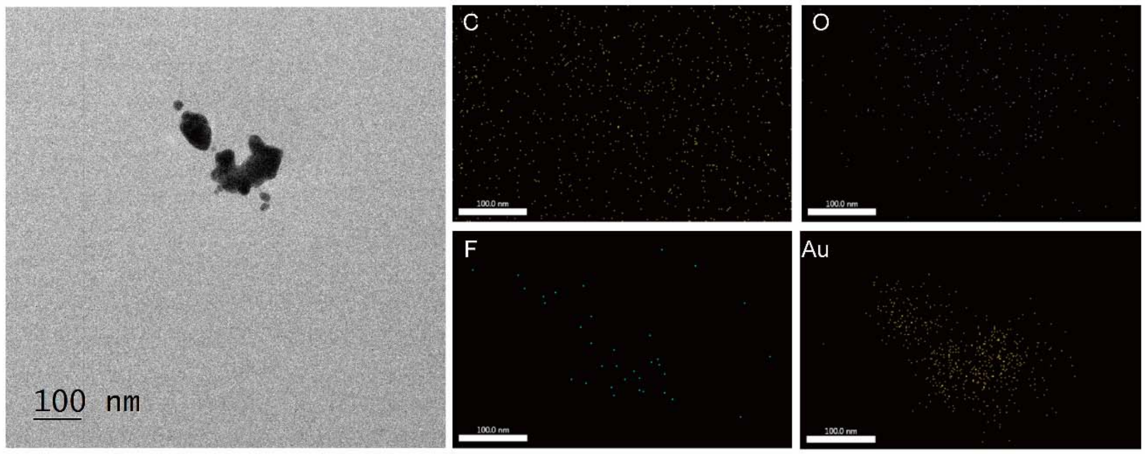


**Supplementary Fig. 15 |** Energy dispersive X-ray spectroscopy images of reduced Au.

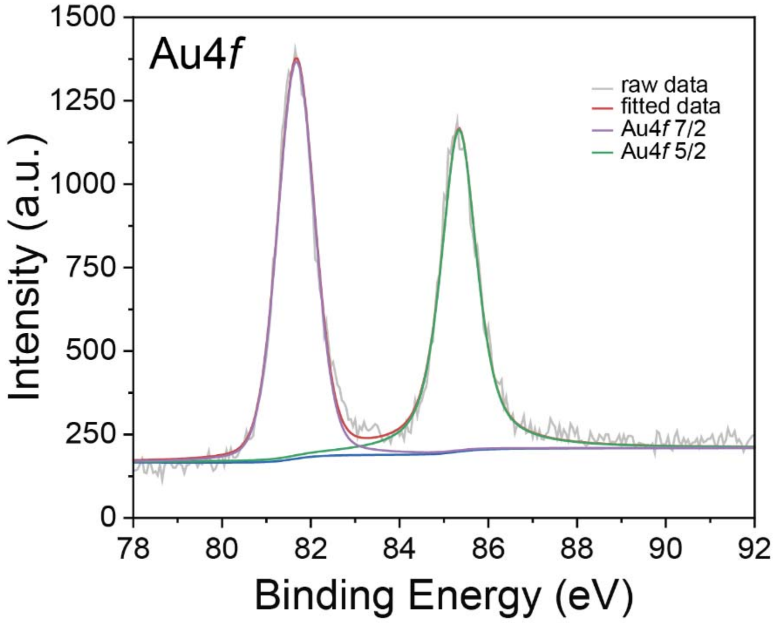


**Supplementary Fig. 16 |** XPS Au4*f* spectra of reduced gold.

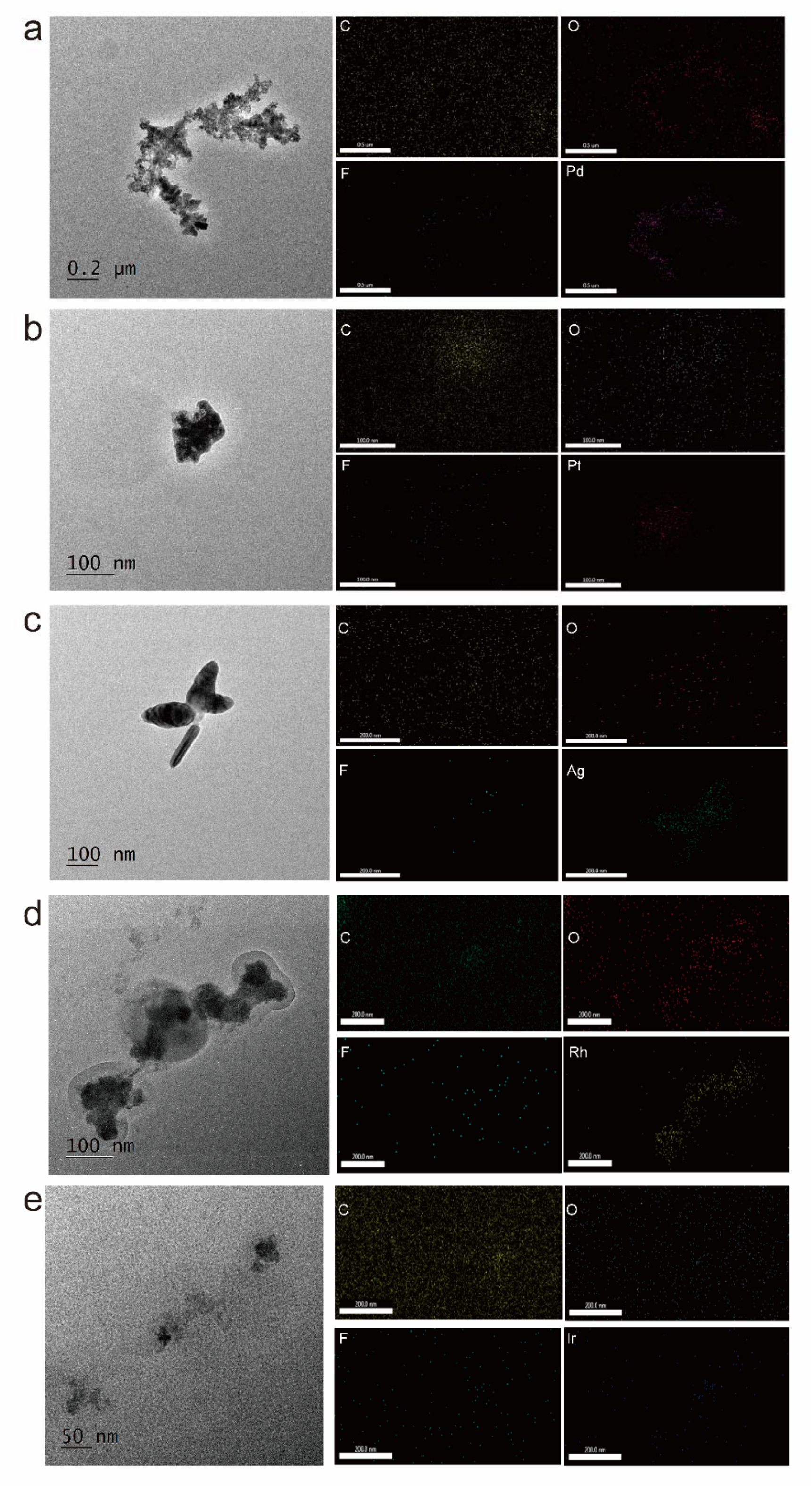


**Supplementary Fig. 17 |** Energy dispersive X-ray spectroscopy images of reduced (a) Pd, (b) Pt, (c) Ag , (d) Rh , and (e) Ir.

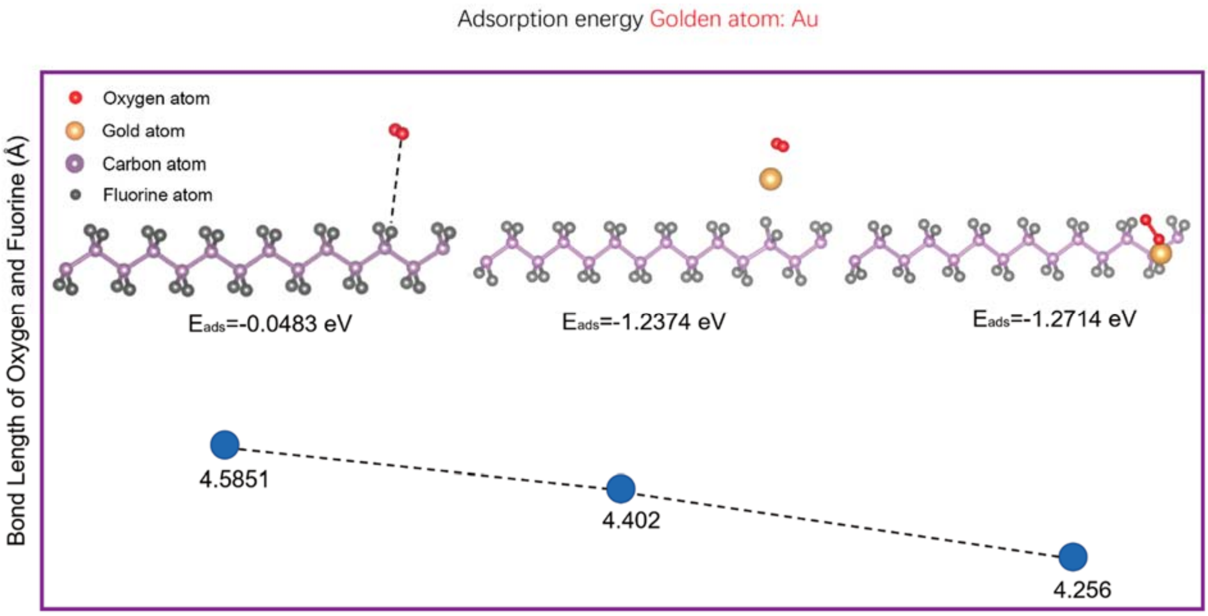


**Supplementary Fig. 18 |** Calculated distance between O of oxygen and F, and adsorption energy for PTFE and $O_2$. Carbon: Purple, Grey: Fluoride, Red: Oxygen, Yellow: Gold.

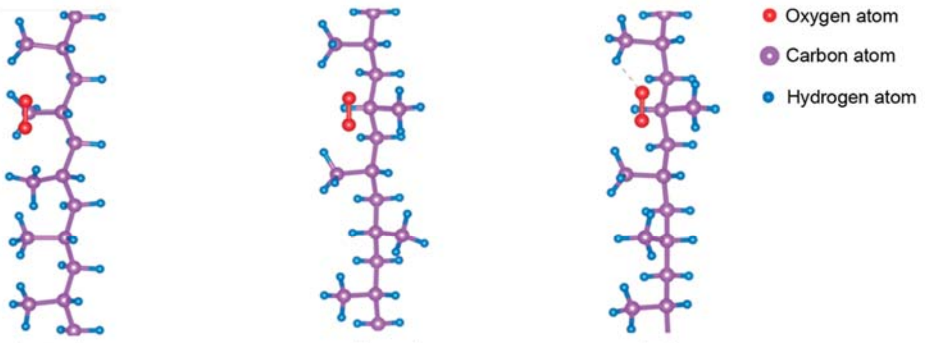


| | isotactic | syndiotactic | atactic |
|---|---|---|---|
| Bond length (Å) | 2.1116 | 2.1116 | 2.1116 |
| Energy difference (eV) | 1.8265 | 1.8297 | 1.8238 |

**Supplementary Fig. 19 |** Simulated energy difference $\Delta E(HOMO_{(PTFE)}- \pi_{(O2)})$ for isotactic, syndiotactic and atactic PP. Carbon: purple, Hydrogen: blue, Oxygen: red.

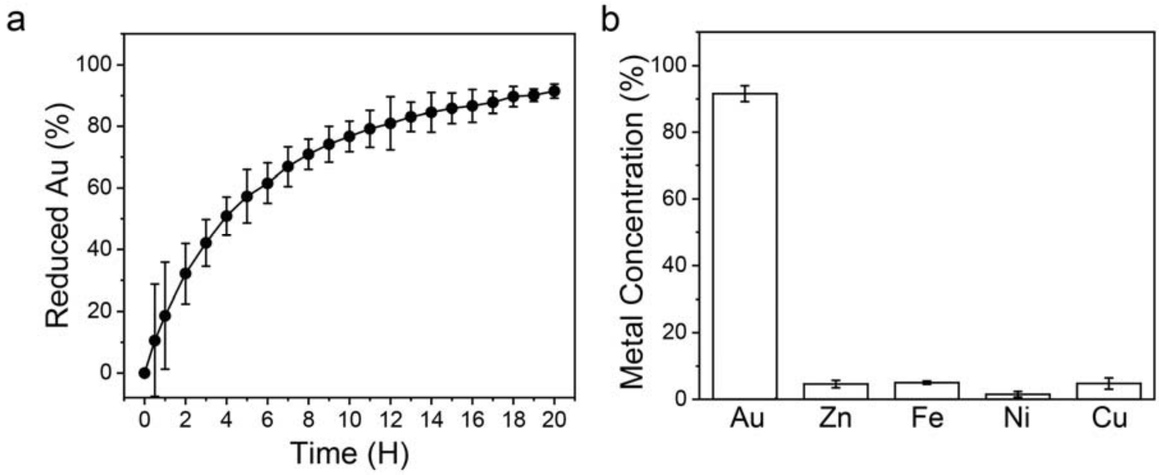


**Supplementary Fig. 20 |** Real world application for electroplating waste. (a) Evolution of Au concentration from electroplating waste. Lines are guide to eyes. (b) Amount of Au and other metals that were extracted from e-waste. For all figures, error bars represent standard deviations for 3 reproduced experiments.

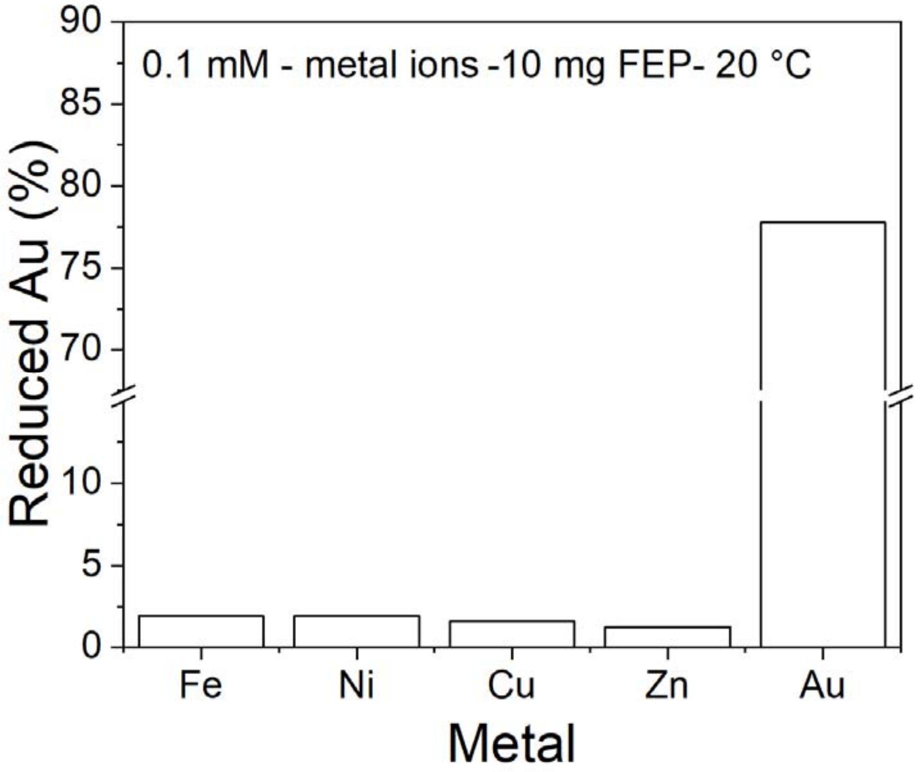


**Supplementary Fig. 21 |** Fe, Ni, Cu, Zn, and Au extraction amount in their anaerobic aqueous solution. The reaction condition is: 0.1 mM concentration for each ion with 10 mg FEP, in anaerobic conditions at 20 °C.

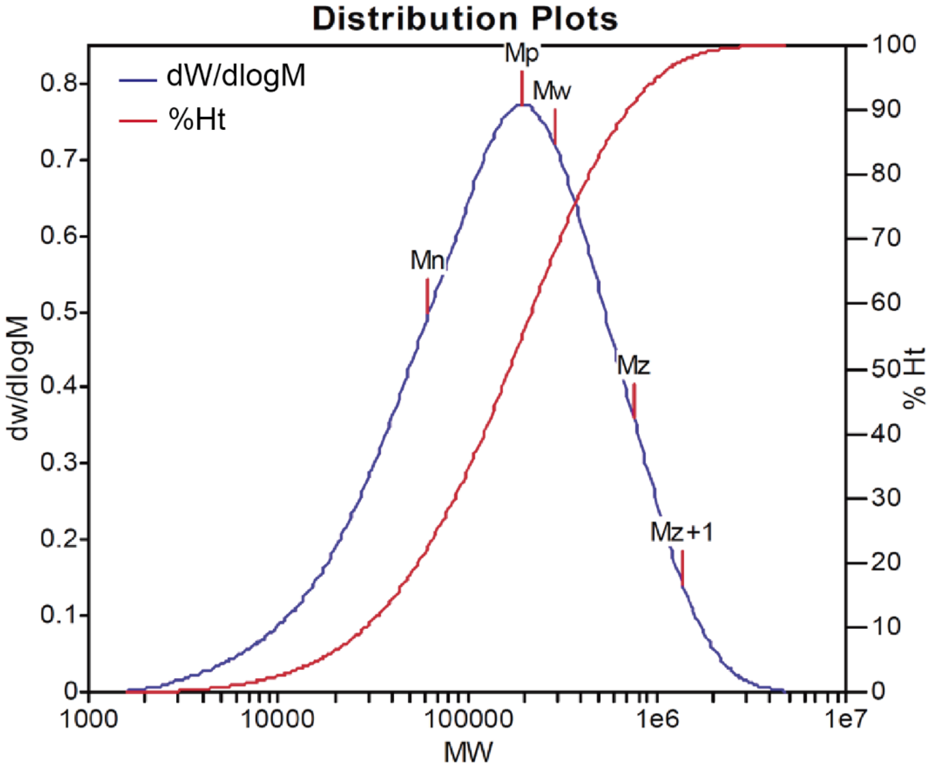


**Supplementary Fig. 22 |** GPC analysis of pristine PP. Mn=62672, Mw=289392, Mz=755130.

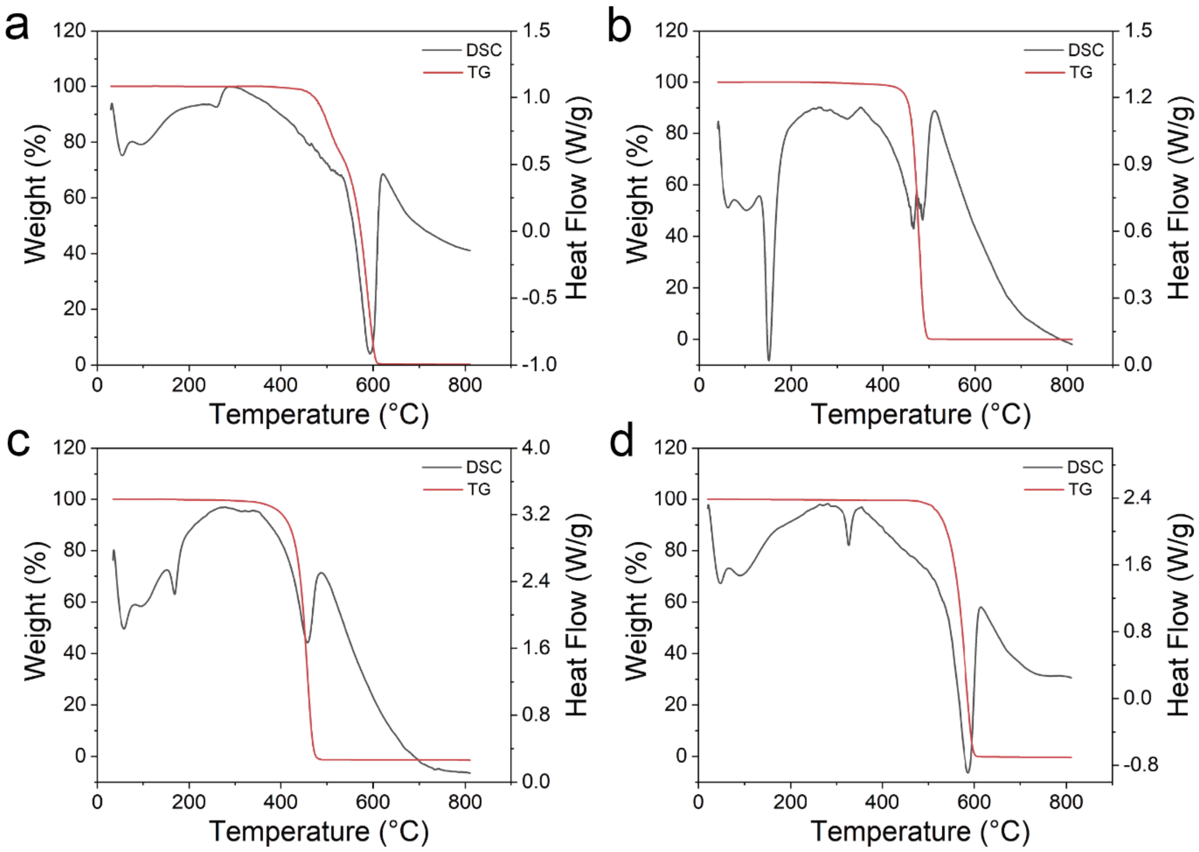


**Supplementary Fig. 23 |** TG-DSC analysis of pristine (a) FEP, (b) HDPE, (c) PP, and (d) PTFE.

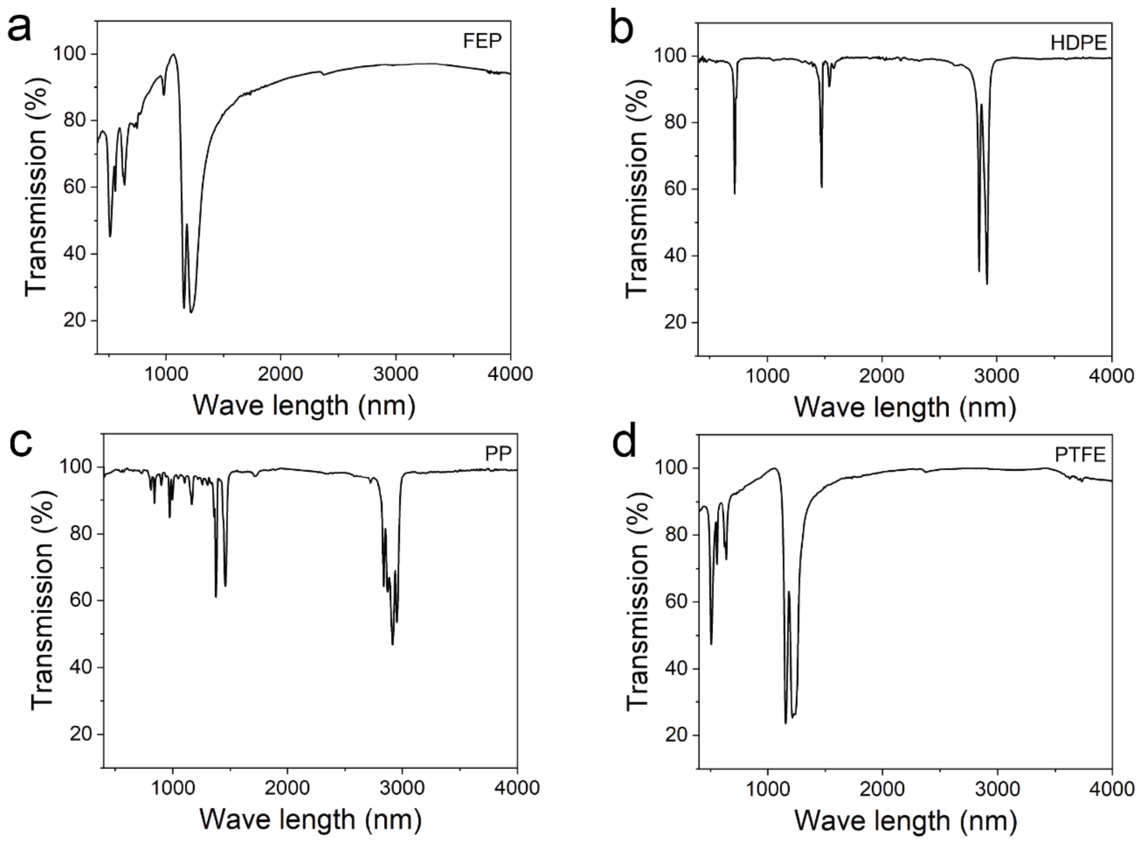


**Supplementary Fig. 24 |** Fourier Transformed Infra-Red Spectroscopy (FTIR) of pristine (a) FEP, (b) HDPE, (c) PP, and (d) PTFE.

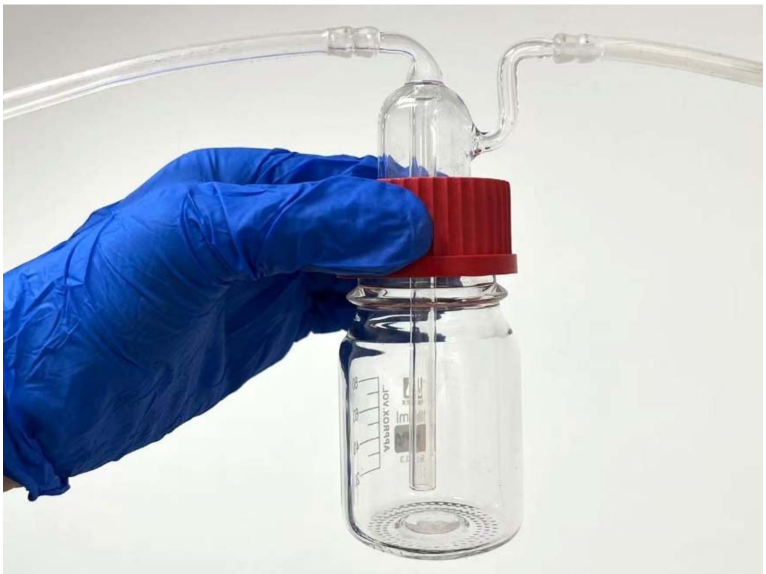

**Supplementary Fig. 25 |** Photograph of reactor. The seal consists of a PTFE gasket, rubber ring and silica gel grommet.

**Supplementary Table 1. Intermittent (Ultrasonication 30 min ON/30 min OFF, with a total ON time of 60 min) and continuous (60 min ON) result for 120 W 40 kHz and 60 W 40 kHz ultrasonication.**

| Time (min) | Gold extraction rate (%) | |
|---|---|---|
| | 40 kHz, 120 W | 40 kHz, 60 W |
| 30 | 44.37197 | 19.68413 |
| 60 | 43.88840 | 19.13771 |
| 90 | 77.65794 | 32.96148 |
| 120 | 77.00936 | 32.39498 |
| 60 (continuous) | 77.75359 | 33.03139 |

**Supplementary Table 2. Surface properties of different sizes of FEP powder.**

| Size (um) | Specific surface area ($m^2$ $g^{-1}$) | Total pore volume ($cm^3$ $g^{-1}$) | Average pore diameter (nm) | Porosity ($\rho$=2.15 $cm^3$ $g^{-1}$) |
|---|---|---|---|---|
| 0.2 | 88.458 | 0.24970 | 11.290 | 0.536877 |
| 2 | 9.023 | 0.02880 | 12.770 | 0.061920 |
| 6.5 | 5.159 | 0.02356 | 18.260 | 0.050654 |
| 15 | 4.017 | 0.01005 | 10.000 | 0.021608 |
| 30 | 3.997 | 0.00990 | 9.906 | 0.021285 |

## Supplementary Table 3. Literature Review.

| [Au] ppm | Types of Au Ion | Method | Material | Extraction Capacity (mg g$^{-1}$) | Extraction time (h) | Reference |
|---|---|---|---|---|---|---|
| 10 | $AuCl_4^-$ | Adsorbent | Fe-BTC/PpPDA | 96 | 0.033 | 9 |
| 20 | | | COP-180 | 100 | 0.5 | 10 |
| 12.5 | | | TDAC | 17.5 | 24 | 11 |
| 100 | | | TDAC | 30 | 24 | |
| 14.8 | $Au(S_2O_3)_2^{3-}$ | | MoS2/CS Aerogel | 50 | 22 | 12 |
| 116 | | | MoS2/CS Aerogel | 600 | 22 | |
| 9.7 | | | MoS2/CS Aerogel | 20 | 22 | |
| 150 | | | UiO-66-TA | 260 | | 13 |
| 80 | | | CSGO5 | 400 | 16 | 14 |
| 100 | $AuCl_4^-$ | | UiO-66 | 60 | 0.41666666 | 15 |
| 100 | | | UiO-66-NH2 | 100 | 3 | |
| 150 | | | UiO-66-TU | 275 | | 16 |
| 100 | | | barley straw carbon | 256 | 1 | 17 |
| 50 | | | L-Lysine modified, crosslinked chitosan resin | 13 | 4 | 18 |
| 40 | | | cross-linked lignocatechol | 40 | 24 | 19 |
| 60 | $Au^{3+}$ | | modified wheat straw | 125 | 24 | 20 |
| 100 | | | CNT-MoS2 | 1000 | 4 | 21 |
| 20 | | | PAF-1-thiourea | 250 | 24 | 22 |
| 100 | $Au(S_2O_3)_2^{3-}$ | | MoS2/ZnS | 1120 | 5 | 23 |
| 50 | | | MsS2/Zns | 500 | 5 | |
| 100 | $AuCl_4^-$ | | rGo nanosheets at 25C | 1880 | 24 | 24 |
| 10 | | | | 1850 | 24 | |
| 1 | | | | 1180 | 24 | |
| 0.1 | | | | 690 | 24 | |
| 0.197 | $AuCl_4^-$ | CEC | FEP | 0.75609 | 3 | This work |
| 1.97 | | | FEP | 9.3872 | 3 | |
| 19.7 | | | FEP | 95 | 3 | |
| 197 | | | FEP | 722.5 | 3 | |

**Supplementary Table 4. D-spacing and SAED analysis of reduced metals.**

| | | | | | | | |
|---|---|---|---|---|---|---|---|
| Au | | d(111) | | Rh | | d(111) | d(111) |
| | HRTEM | 2.35 | | | HRTEM | 2.2 | 2.19 |
| | SAED | 2.35 | | | SAED | 2.2 | |
| | PDF#04-0784 | 2.355 | | | PDF#04-0784 | 2.196 | |
| Ir | | d(111) | d(111) | Pt | | d(111） | d(111) |
| | HRTEM | 2.226 | 2.231 | | HRTEM | 2.12 | 2.135 |
| | SAED | 2.22 | | | SAED | 2.27 | |
| | PDF#06-0598 | 20.33 | | | PDF#04-0802 | 2.26 | |
| Pd | | d(111) | | Ag | | d(111) | |
| | HRTEM | 2.219 | | | HRTEM | 2.397 | |
| | SAED | 2.25 | | | SAED | 2.36 | |
| | PDF#46-1043 | 2.246 | | | PDF#04-0784 | 2.359 | |

# Supplementary References


1. Lin, S., Chen, X. & Wang, Z. L. Contact Electrification at the Liquid–Solid Interface. *Chem. Rev.* **122**, 5209–5232 (2022).
2. Lin, S., Xu, C., Xu, L. & Wang, Z. L. The Overlapped Electron-Cloud Model for Electron Transfer in Contact Electrification. *Adv. Funct. Mater.* **30**, 1909724 (2020).
3. Berbille, A. *et al.* Mechanism for Generating H2O2 at Water-Solid Interface by Contact-Electrification. *Adv. Mater.* **n/a**, 2304387.
4. Zhao, Y. *et al.* The process of free radical generation in contact electrification at solid-liquid interface. *Nano Energy* **112**, 108464 (2023).
5. Song, W.-Z. *et al.* Insulator polymers achieve efficient catalysis under visible light due to contact electrification. *Water Res.* **226**, 119242 (2022).
6. Wang, Z. *et al.* Contact-electro-catalysis for the degradation of organic pollutants using pristine dielectric powders. *Nat. Commun.* **13**, 1–9 (2022).
7. Vogel, Y. B. *et al.* The corona of a surface bubble promotes electrochemical reactions. *Nat. Commun.* **11**, 6323 (2020).
8. Chen, B. *et al.* Water–solid contact electrification causes hydrogen peroxide production from hydroxyl radical recombination in sprayed microdroplets. *Proc. Natl. Acad. Sci.* **119**, e2209056119 (2022).
9. Sun, D. T., Gasilova, N., Yang, S., Oveisi, E. & Queen, W. L. Rapid, Selective Extraction of Trace Amounts of Gold from Complex Water Mixtures with a Metal–Organic Framework (MOF)/Polymer Composite. *J. Am. Chem. Soc.* **140**, 16697–16703 (2018).
10. Hong, Y. *et al.* Precious metal recovery from electronic waste by a porous porphyrin polymer. *Proc. Natl. Acad. Sci.* **117**, 16174–16180 (2020).

11. Dwivedi, A. D., Dubey, S. P., Hokkanen, S., Fallah, R. N. & Sillanpää, M. Recovery of gold from aqueous solutions by taurine modified cellulose: An adsorptive–reduction pathway. *Chem. Eng. J.* **255**, 97–106 (2014).

12. Chen, P., Liang, Y., Yang, B., Jia, F. & Song, S. In Situ Reduction of Au(I) for Efficient Recovery of Gold from Thiosulfate Solution by the 3D MoS2/Chitosan Aerogel. *ACS Sustain. Chem. Eng.* **8**, 3673–3680 (2020).

13. Wang, C. *et al.* Highly selective recovery of Au(III) from wastewater by thioctic acid modified Zr-MOF: Experiment and DFT calculation. *Chem. Eng. J.* **380**, 122511 (2020).

14. Liu, L. *et al.* Preparation and characterization of chitosan/graphene oxide composites for the adsorption of Au(III) and Pd(II). *Talanta* **93**, 350–357 (2012).

15. Lin, S. *et al.* Effective adsorption of Pd(II), Pt(IV) and Au(III) by Zr(IV)-based metal–organic frameworks from strongly acidic solutions. *J. Mater. Chem. A* **5**, 13557–13564 (2017).

16. Wu, C. *et al.* Specific Recovery and In Situ Reduction of Precious Metals from Waste To Create MOF Composites with Immobilized Nanoclusters. *Ind. Eng. Chem. Res.* **56**, 13975–13982 (2017).

17. Chand, R. *et al.* Selective adsorption of precious metals from hydrochloric acid solutions using porous carbon prepared from barley straw and rice husk. *Miner. Eng.* **22**, 1277–1282 (2009).

18. Fujiwara, K., Ramesh, A., Maki, T., Hasegawa, H. & Ueda, K. Adsorption of platinum (IV), palladium (II) and gold (III) from aqueous solutions onto l-lysine modified crosslinked chitosan resin. *J. Hazard. Mater.* **146**, 39–50 (2007).

19. Parajuli, D. *et al.* Selective Recovery of Gold by Novel Lignin-Based Adsorption Gels. *Ind. Eng. Chem. Res.* **45**, 8–14 (2006).

20. Wang, J., Li, J. & Wei, J. Adsorption characteristics of noble metal ions onto modified straw bearing amine and thiol groups. *J. Mater. Chem. A* **3**, 18163–18170 (2015).

21. Liu, F., You, S., Wang, Z. & Liu, Y. Redox-Active Nanohybrid Filter for Selective Recovery of Gold from Water. *ACS EST Eng.* **1**, 1342–1350 (2021).

22. Ma, T. *et al.* Efficient Gold Recovery from E-Waste via a Chelate-Containing Porous Aromatic Framework. *ACS Appl. Mater. Interfaces* **12**, 30474–30482 (2020).

23. Zhan, W., Yuan, Y., Yang, B., Jia, F. & Song, S. Construction of MoS2 nano-heterojunction via ZnS doping for enhancing in-situ photocatalytic reduction of gold thiosulfate complex. *Chem. Eng. J.* **394**, 124866 (2020).

24. Li, F. *et al.* Highly efficient and selective extraction of gold by reduced graphene oxide. *Nat. Commun.* **13**, 4472 (2022).
